# Role of metastasectomy in the management of renal cell carcinoma

**DOI:** 10.3389/fsurg.2022.943604

**Published:** 2022-07-29

**Authors:** Mark Mikhail, Kevin J. Chua, Labeeqa Khizir, Alexandra Tabakin, Eric A. Singer

**Affiliations:** Section of Urologic Oncology, Rutgers Cancer Institute of New Jersey and Rutgers Robert Wood Johnson Medical School, New Brunswick, NJ, United States

**Keywords:** Metastatic kidney cancer, metastasectomy, renal cell carcinoma, metastatic renal cell carcinoma, renal cancer

## Abstract

Treatment of metastatic renal cell carcinoma (mRCC) has evolved with the development of a variety of systemic agents; however, these therapies alone rarely lead to a complete response. Complete consolidative surgery with surgical metastasectomy has been associated with improved survival outcomes in well-selected patients in previous reports. No randomized control trial exists to determine the effectiveness of metastasectomy. Therefore, reviewing observational studies is important to best determine which patients are most appropriate for metastasectomy for mRCC and if such treatment continues to be effective with the development of new systemic therapies such as immunotherapy. In this narrative review, we discuss the indications for metastasectomies, outcomes, factors associated with improved survival, and special considerations such as location of metastasis, number of metastases, synchronous metastases, and use of systemic therapy. Additionally, alternative treatment options and trials involving metastasectomy will be reviewed.

## Introduction

Renal cell carcinoma (RCC) is a life-threatening malignancy with gradually increasing incidence worldwide, accounting for 79,000 new cases in the United States (1). Renal cancer is the 6th and 9th most common cancer in males and females, respectively, and led to 13,920 estimated deaths in 2022 (1). RCC is the ninth most common neoplasm in the United States overall, which has led to rapid development in treatment approaches over the past decade.

While localized RCC can be removed surgically, up to 17% of cases are metastatic at the time of diagnosis. Furthermore, 20%–40% of patients with localized disease who initially undergo extirpative surgical treatment will eventually develop distant metastasis (2). Systemic therapy is the mainstay of treatment in advanced renal cancer and options have expanded to include a range of new drugs and combination therapies (Table 1) (3–8). Recently, combination regimens including doublet immunotherapy and immunotherapy with tyrosine kinase inhibitor therapy have been developed. For instance, axitinib and pembrolizumab combination therapy (approved in 2019), resulted in a superior overall response rate and progression-free survival (PFS) compared to sunitinib (8, 9). Regardless of the evolution in targeted therapy, complete response (CR) and cure for mRCC through systemic treatment alone is rare. For instance, interleukin-2 had a CR rate of 5.4% according to the PROCLAIM registry (10). Antiangiogenic therapy showed a CR rate of 2%, while recent randomized controlled trials (RCTs) demonstrated a CR rate of 3%–5% for sunitinib (11–14). Meanwhile, immunotherapy CR ranged from 1%–7% in PD-L1 negative patients and 6%–16% for PD-L1 positive patients.^13^Given the poor CR rate of systemic therapy, there is room for improvement for treatment of advanced renal cancer. Previous studies have shown that complete resection of the primary tumor for mRCC is associated with improved outcomes in properly selected patients (15). For instance, Singla et al. demonstrated improved overall survival for patients with metastatic clear cell RCC for those undergoing a cytoreductive nephrectomy (CN) with immunotherapy treatment versus immunotherapy alone (16). Furthermore, it has been shown that metastasectomy can also offer durable survival benefit (17, 18). Surgical resection may be beneficial given that bulky tumors can inhibit immune responses that are vital to combating cancer (19). Large primary tumors may lead to the repression of T-cell function, and prior studies have demonstrated the inability of systemic agents to generate significant responses in primary tumors of mRCC patients (19). Although there are no randomized controlled trials that have assessed the benefit of surgical metastasectomy in mRCC, there remains a large body of observational studies that account for the benefit of using this approach (20). In this narrative review, we aim to discuss the indications, outcomes, factors associated with improved survival, and use of systemic therapy for metastasectomy in mRCC. Additionally, alternative treatment options and trials involving metastasectomy will be reviewed.

**Table 1:** FDA-Approved systemic therapies for the treatment of metastatic renal cell carcinoma.

Therapy	FDA Approval	Treatment line	Mechanism of action	Route	Comparator arm	Primary endpoint
Interleukin-2	May-92	First	Cytokine immunotherapy	IV	Phase II- none	ORR
Sorafenib	Dec-05	Cytokine failure	VEGFR, PDGFR, RET, KIT Inhibitor	Oral	Placebo	OS
Sunitinib	Jan-06	First	VEGFR, PDGFR inhibitor	Oral	IFN-α	PFS
Temsirolimus	May-07	First	mTOR inhibitor	IV	IFN-α	OS
Everolimus	Mar-09	VEGFR failure	mTOR inhibitor	Oral	Placebo	PFS
Bevacizumab + IFN-α	Jul-09	First	Anti-VEGF monoclonal antibody	IV + SC	IFN-α ± placebo	OS
Pazopanib	Oct-09	First or cytokine failure	VEGFR, PDGFR, KIT inhibitor	Oral	Placebo	PFS
Axitinib	Jan-12	Second	VEGFR inhibitor	Oral	Sorafenib	PFS
Nivolumab	Nov-15	Second	Anti-PD1 monoclonal antibody	IV	Everolimus	OS
Cabozanitinib	Apr-16	Second	VEGFR, MET, AXL inhibitor	Oral	Everolimus	PFS
Lenvatinib + Everolimus	May-16	Second	VEGFR, FGFR, PDGFR, RET, KIT inhibitor, mTOR inhibitor	Oral	Everolimus or Lenvatinib	PFS
Axitinib + Pembrolizumab	Apr-19	First	VEGFR, anti-PD1 monoclonal antibody	Oral (Axitinib),	Sunitinib	ORR, PFS
				IV (Pembrolizumab)		
Cabozanitinib + Nivolumab	Jan-21	First or cytokine failure	VEGFR, MET, AXL inhibitor, Anti-PD1 monoclonal antibody	Oral (Cabozanitinib),	Sunitinib	ORR, PFS
				IV (Nivolumab)		
Lenvatinib + Pembrolizumab	Aug-21	First	VEGFR, FGFR, PDGFR, Anti-PD1 monoclonal antibody	Oral (Lenvatinib),	Sunitinib or Lenvatinib/Everolimus	ORR, PFS
				IV (Pembrolizumab)		
Ipilimumab + Nivolumab	Apr-18	First	Anti-CTLA-4 monoclonal antibody, Anti-PD1 monoclonal antibody	IV	Sunitinib	ORR, OS, PFS
Axitinib + Avelumab	May-19	First	VEGFR inhibitor, Anti-PDL1 monoclonal antibody	IV (Avelumab),	Sunitinib	OS
				oral (Axitinib)		
Tivozanib	Mar-21	Subsequent	TKI	Oral	Sorafenib	PFS

IV, intravenous; ORR, objective response rate; OS, overall survival; PFS, progression-free survival; SC, subcutaneous.

Adapted and modified from Shinder, B. M., Rhee, K., Farrell, D. et al. Surgical Management of Advanced and Metastatic Renal Cell Carcinoma: A Multidisciplinary Approach. Front Oncol, 7: 107, 2017. This is an open access article distributed under the Creative Commons Attribution License (https://creativecommons.org/licenses/by/4.0/).

## Methods

A literature search using MEDLINE and Web of Science was done to perform a comprehensive non-systematic review of articles using the search terms “renal cell carcinoma”, “renal cancer”, “kidney cancer”, “metastasectomy”, “metastectomy”, “resection”, and “synchronous” to identify studies involving metastasectomies for mRCC. Articles selected were required to be original articles written in English. Systematic reviews, original articles, and case reports/series were included. Commentaries and news articles were excluded. The studies were independently reviewed. References of papers were reviewed for potential missed studies.

Information on clinical trials was collected from www.clinicaltrials.gov, which was accessed in March 2022. Trials were selected by using combinations of the search terms “metastasectomy”, “renal cell carcinoma”, “kidney cancer”, “renal cell cancer”, “kidney”, “resection” and “metastatic renal cell carcinoma”. Trials were classified as completed if their status was listed as “completed” on www.clinicaltrials.gov. Trials were classified as ongoing if their status was listed as “active”, “recruiting”, “active, not yet recruiting”, “active, not recruiting”, or “suspended”. Trials were also identified for being “terminated” or “withdrawn.”

## Metastasectomy status and clinical outcomes

Many retrospective series have been published regarding the efficacy of metastasectomy in mRCC. Studies that reported the difference in clinical outcomes between patients who received complete metastasectomy (MTX), those who were treated with an incomplete metastasectomy (iMTX), or those who did not receive metastasectomy (non-MTX) were identified and reviewed.

When comparing MTX to non-MTX, many studies found that MTX patients had significantly improved overall survival (OS) (21–40). These findings were consistent after groups were propensity score-matched (PSM) for characteristics such as synchronous metastasis, time from diagnosis of RCC to metastasis, pathological T stage, site of metastasis, and clear cell histology (21–23, 38). In 2020, Dragomir et al. conducted a 1:4 PSM analysis of 229 MTX patients and found that the median OS was significantly different compared to the matched group (81 months (interquartile range [IQR] 58 – Not reached (NR)) vs. 61 months (IQR 26 – NR; *p* = 0.0001), respectively (21). Similarly, MTX was also associated with improved cancer-specific mortality (CSM) and PFS (24, 28, 32, 38, 41, 42). A large study by Wu et al. in 2020 utilizing data from 2,911 mRCC patients found that MTX was significantly associated with decreased CSM (3-year cumulative incidence 52.6 vs. 59.2%, Hazard Ratio (HR) 0.875, 95% Confidence Interval (CI) 0.773–0.991; *p* = 0.015) (24).

Several studies demonstrated that certain patient populations had a lack of clinical improvement with metastasectomy for mRCC. Fares et al. compared 37 MTX patients with 37 PSM non-MTX patients and found that the OS benefit of MTX was absent in patients with poor International Metastatic RCC Database Consortium (IMDC) risk stratification (33). Similarly, while Wu et al. demonstrated a significant decrease in CSM overall, a sub-analysis revealed that only IMDC favorable-risk patients benefitted from MTX, whereas no significant difference was observed for intermediate and high-risk patients (24). In 2010, Staehler et al. studied 88 mRCC patients with isolated liver metastasis and demonstrated that MTX patients with Fuhrman grade 3–4 primary RCC or patients with synchronous metastasis did not benefit from surgery (43).

iMTX compared to MTX and non-MTX for mRCC has also been examined. You et al. demonstrated that for patients receiving targeted therapy, median PFS was 29.5, 18.8, and 14.8 months (*p* < 0.001) for MTX (*n* = 33), iMTX (*n* = 29), and non-MTX (*n* = 263) patients, respectively (28). A 2021 study by Ishihara et al. comparing the three groups revealed a relationship between metastasectomy status and OS (29). Median OS were NR, 81.5 months (*p* = 0.0042) and 28.1 months (*p* < 0.0001) for MTX (*n* = 45), iMTX (*n* = 53), and non-MTX (*n* = 216) patients, respectively. The study also found that the iMTX group had a significantly longer OS than non-MTX (*p* = 0.0010). These results have been corroborated by similar findings in several other studies (40, 44–48).

Another critical factor that must be considered is the presence of oligometastatic versus polymetastatic disease in determining which patients will benefit from MTX. While no studies specifically defined a cut-off for the number of metastases between these two groups, multiple studies have examined the prognostic role that number of metastases has in this patient population (32, 36, 38, 46, 49, 50). In 2011, Alt. et al reported on 887 mRCC patients who developed multiple metastases, of whom 125 (14%) underwent MTX (38). This study demonstrated that complete MTX was more likely in patients with 2 metastases versus ≥3 metastases (*p* < 0.001). Another study in 2011 by Meimarakis et al., which reported on clinical outcomes in 202 mRCC patients with pulmonary metastases, demonstrated that median survival was significantly better in patients with <3 metastases versus ≥3 metastases (56.6 months, 95% CI, 33.5–79.8 vs. 29.9 months, 95% CI, 26.3–33.4; *p* = 0.011) (46). A 2002 study by Piltz et al. reported on clinical outcomes for 105 mRCC patients with pulmonary metastases who underwent MTX, and found that patients with >2 metastases had significantly worse overall survival rates versus those with ≤2 metastases (*p* = 0.029) (36). The data demonstrates that clinicians should also weigh the number of metastases in determining whether patients should undergo metastasectomy.

Taken together, most studies demonstrate that completeness of metastasectomy is associated with improved clinical outcomes; however, it is important to note that these retrospective studies are subject to selection bias. Many patients who do undergo MTX may be selected from the population based on their health status, if the surgeon deems a patient fit for surgery or if a metastatic lesion is resectable. For instance, Russo et al. demonstrated that in 91 patients with synchronous mRCC who underwent CN, median survival was 30 months versus 12 months for those who did and did not undergo metastasectomy, respectively (20). The authors cautioned that this difference in survival likely indicated a more limited extent of disease in those who were able to have a metastasectomy performed. Such factors must be considered when evaluating the data presented. However, even with such factors at play, multiple studies have utilized PSM to control for these variables and still demonstrated improved outcomes in patients who undergo MTX (21–23, 38). Despite this, randomized controlled trials are needed to better understand the survival benefits of metastasectomy.

## Clinical outcomes and complications of metastasectomy by site of disease

### Lung

Among the various sites of metastasis for mRCC metastasectomy, lung metastases were the most reported in the literature. In a study of the National Inpatient Sample (NIS) database published in 2017, Meyer et al. reported on 45,279 mRCC patients and found that metastatic disease to the lungs was most common (52% overall), followed by bone, liver, lymph nodes, adrenal glands, and brain (51). In 2017, Zhao et al. published a meta-analysis of 1,447 patients with mRCC who underwent lung MTX and reported pooled 1, 3, and 5-year OS of 84%, 59%, and 43%, respectively (18). This study also reported that incomplete resection of metastases was associated with worse survival (HR 3.74, 95% CI, 2.49–5.61; *p* = 0.000).

In 2018, Sun et al. published a large retrospective study of 6,994 mRCC patients, of whom 1,976 underwent MTX (22). They found significant survival differences at 1, 2, and 3 years for patients with lung metastases that underwent MTX versus those who did not undergo MTX (77.9%, 58.9%, and 47.3% vs. 65.2%, 44.9%, and 34.4%; *p* = 0.003). Likewise, on multivariate analysis, lung MTX was associated with a significantly lower hazard of death or any death rate versus non-MTX (HR 0.67, 95% CI, 0.51–0.88; *p* = 0.004).

Numerous studies have published data on prognostic factors identified *via* multivariate analysis in mRCC patients with lung metastases, which include synchronous metastasis, completeness of metastasectomy, and number of metastases (Table 2) (36, 46, 52–63). In 2021, Meacci et al. published the results of a multi-institutional study on 210 mRCC patients who underwent lung MTX. One of the strongest prognosticators for OS identified was <80% on the Karnofsky Performance Status Scale (KPSS), (HR 24.381, 95% CI, 3.842–154.700; *p *< 0.001). KPSS was also a strong prognostic factor for disease-free interval (DFI), (HR 18.104, 95% CI, 2.090–156.849; *p* = 0.009), and disease-free survival (DFS), (HR 15.649, 95% CI, 2.992–81.851; *p* = 0.001) (55). Meacci et al. also identified synchronous metastasis (OS HR 2.934, 95% CI, 1.324–6.505; *p* = 0.008), non-clear-cell histology (DFS HR 3.475, 95% CI, 1.239–9.748; *p* = 0.018), and the presence of multiple lung metastases (DFS HR 1.721, 95% CI, 1.036–2.858; *p* = 0.036) as poor prognosticators. A 2020 study by Holz et al. of 138 mRCC patients with lung metastases (62) identified the presence of nonpulmonary metastasis (OS HR 2.29, IQR 1.02–5.10; *p* = 0.0449) and sarcomatoid dedifferentiation (OS HR 4.52, IQR 1.15–17.69; *p* = 0.0313) as poor prognosticators (62).

**Table 2:** Prognostic factors identified *via* multivariate analysis for metastatic renal cell carcinoma in patients with lung metastases.

Study	Year	Study Description	Patient #	Prognostic Factors	Endpoint HR/OR/RR (95% CI)	*p*-value
Piltz et al.	2002	Retrospective, Single-center	105	Lymph node status at primary resection	OS OR 1.61 (1.0–2.59)	0.049
				Size of metastasis	OS OR 2.42 (1.40–4.20)	0.0016
Pfannschmidt et al.	2002	Retrospective, Single-center	191	Number of metastases	OS Not Recorded	0.0002
				Complete resection	OS Not Recorded	0.049
				Lymph node metastases	OS Not Recorded	0.0038
				DFI	OS Not Recorded	0.012
Marulli et al.	2006	Retrospective, Single-center	59	Age >60 years	OS Not Recorded	0.02
Assouad et al.	2007	Retrospective, Multi-center	65	Size of lung metastasis	OS Not Recorded	0.0018
				Lymph node involvement	OS Not Recorded	0.0018
Winter et al.	2010	Retrospective,Single -center	110	Completeness of metastasectomy	OS HR 5.1 (2.2–11.8)	<0.001
				pN of primary tumor	OS HR 7.2 (3.3–15.6)	<0.001
				Mediastinal/hilar lymph node status	OS HR 5.4 (2.6–11.4)	<0.001
				pN of primary tumor^a^	OS HR 5.7 (2.4–13.3)	<0.001
				Mediastinal/hilar lymph node status^a^	OS HR 5.8 (2.5–13.3)	<0.001
				Pleural infiltration^a^	OS HR 7.1 (1.5–34.2)	<0.001
Meimarakis et al.	2011	Retrospective, Single-center	202	pN1 of primary tumor	OS HR 3.8 (2.2–6.5)	<0.001
				Incomplete resection of metastases	OS HR 3.3 (1.9–5.7)	<0.001
				≥3 metastases	OS HR 1.5 (1.0–2.3)	0.039
				Size of metastasis ≥3 cm	OS HR 1.9 (1.2–3.2)	0.010
				Mediastinal lymph node status	OS HR 3.6 (1.5–8.4)	0.004
				Pleural infiltration^a^	OS HR 5.3 (2.2–12.8)	<0.001
				Synchronous metastasis^a^	OS HR 1.9 (1.2–3.0)	0.009
				pN1 of primary tumor^a^	OS HR 3.0 (1.6–5.5)	<0.001
				Size of metastasis ≥3 cm^a^	OS HR 2.3 (1.3–4.1)	0.005
				Mediastinal lymph node status^a^	OS HR 4.5 (1.7–11.6)	0.002
Kanzaki et al.	2011	Retrospective, Single-center	48	DFI <2 years	OS RR 2.77 (1.31–5.87)	0.01
				Completeness of metastasectomy	OS RR 2.78 (1.03–7.48)	0.04
Kawashima et al.	2011	Retrospective, Single-center	25	Resectability of pulmonary metastases	PFS HR 0.192 (0.030–0.695)	0.012
Bölükbas et al.	2012	Retrospective, Single-center	107	Lymph node status of primary tumor	OS Not Recorded	<0.0001
				Primary tumor grade	OS Not Recorded	0.004
Kudelin et al.	2013	Retrospective, Single-center	116	Age <70 years	OS Not Recorded	0.005
Renaud et al.	2014	Retrospective, Multi-center	122	Absence of nodal involvement	OS HR 0.384 (0.179–0.825)	0.01
				DFI ≤12 months	OS HR 3.081 (1.193–7.957)	0.02
					RFS HR 2.529 (1.403–4.557)	0.002
				Charlson Comorbidity Index0 vs. 2	OS HR 0.053 (0.009–0.310)	0.01
				Age at metastasectomy ≤60 years	RFS HR 9.657 (4.922–18.944)	<0.0001
Baier et al.	2015	Retrospective, Single-center	237	iMTX	OS HR 2.4 (1.84–3.13)	<0.0001
				≥10 metastases	OS HR 1.27 (0.81–1.81)	0.0029
Holz et al.	2020	Retrospective, Single-center	138	pT stage >2	OS HR 2.79 (1.47–5.28)^b^	0.0017
				No evidence of disease not reached	OS HR 8.62 (3.19–23.32)^b^	<0.0001
				Nonpulmonary metastasis	OS HR 2.29 (1.02–5.10)^b^	0.0449
				Sarcomatoid dedifferentiation	OS HR 4.52 (1.15–17.69)^b^	0.0313
Meacci et al.	2021	Retrospective, Multi-center	210		OS HR 2.934 (1.324–6.505)	0.008
				Synchronous metastasis	DFI HR 1.899 (1.032–3.495)	0.039
				Male Gender	OS HR 24.381 (3.842–154.700)	<0.001
				KPSS <80%	DFI HR 18.104 (2.090–156.849)	0.009
				LDH >1.5 times 140 U/l	DFS HR 15.649 (2.992–81.851)	0.001
				Non-clear-cell histology	DFI HR 3.385 (1.659–6.906)	0.001
				Multiple lung metastases	DFS HR 3.476 (1.239–9.748)	0.018
					DFS HR 1.721 (1.036–2.858)	0.036

OS, overall survival; OR, odds ratio; HR, hazard ratio; RR, relative risk; PFS, progression-free survival; DFI, disease-free interval; DFS, disease-free survival.

^a^ For patients who underwent complete metastasectomy.

^b^ Interquartile range; iMTX, incomplete metastasectomy; KPSS, Karnofsky performance status scale; LDH, lactate dehydrogenase.

With regards to post-operative complications, Meyer et al. reported that patients undergoing lung MTX were significantly less likely to have any complications on univariate analysis compared to MTX of any other site (Odds Ratio (OR) = 0.63, 95% CI, 0.50–0.81, *p *<* *0.001) (51). Lung MTX-reported complications include pneumonia, wound infection, sepsis, arrhythmias, pneumothorax and bronchial stump fistula (36, 45, 54, 64). A study of 105 lung MTX patients conducted by Piltz et al. in 2002 observed complications in 16 of 150 procedures (10.7%) (36). One patient (1.0%) died from severe sepsis after a lower bi-lobectomy. In 2013, Kudelin et al. reported on 116 consecutive lung MTX and standardized intrathoracic lymph node dissection patients, 16 (15%) of whom had post-operative complications, including pneumonia (*n* = 8) and supraventricular arrhythmia (*n* = 3), among others (54). One patient (0.9%) died from pneumonia with sepsis (45, 64). mRCC with lung metastasis has a robust base of studies demonstrating a survival benefit for well-selected patients treated with metastasectomy, but they must also be counseled on possible complications.

### Bone

Bone is the 2nd most common site of metastasis, occurring in 29% of mRCC cases (51). The majority of mRCC patients with bone metastases also have concomitant metastases to other organ sites (50, 65–67). A 2019 study by Ruatta et al. of 300 patients with mRCC and bone metastases reported that only 64 patients (21%) had isolated bone metastases, while the remaining 236 patients (79%) had concomitant metastases to other sites (68).

Surgical management of bone metastases can be categorized into 3 primary groups, namely, curative resection with intent to completely resect all metastases (MTX), intralesional curettage with intent to remove gross tumor but not completely (iMTX), or stabilization surgery with no intent to resect metastases ± radiation to the metastatic site (non-MTX). Patients who do not undergo any surgical intervention are also included in the non-MTX group. In 2014, Hwang et al. reported on 135 patients who underwent curative MTX of bone metastases for mRCC and observed 1-, 3-, and 5-year OS rates of 72%, 45%, and 28%, respectively (50). Another study by Lin et al. in 2007 reported on 295 consecutive mRCC patients who required surgical management (MTX, iMTX, or non-MTX) of appendicular skeletal bone metastases and reported 1-, 2-, and 5-year OS rates of 47%, 30%, and 11%, respectively (65).

The clinical outcomes associated with MTX versus iMTX/non-MTX have also been reported. In 2016, Langerhuizen et al. reported on 183 mRCC patients with bone metastases who underwent surgical treatment with MTX (*n* = 88, 48%), iMTX (*n* = 54, 30%), or non-MTX (*n* = 41, 22%) (67). They observed a significant difference in survival for MTX patients versus iMTX or non-MTX (*p* = 0.020), but that difference was no longer observed when focusing on patients with solitary bone metastasis (*p* = 0.997) or patients with multiple bone metastases (*p* = 0.099). In 2019, Kim et al. reported on 117 mRCC patients with bone metastases and found that both PFS and OS were significantly improved in MTX versus non-MTX patients (32). Median PFS in the MTX and non-MTX groups were 17.79 months (95% CI, 13.74–24.82) and 8.71 months (95% CI, 5.82–10.85; *p* = 0.009), respectively, while median OS in the MTX and non-MTX groups were 31.89 months (95% CI, 21.96–38.50) and 9.65 months (95% CI, 7.40–15.39; *p *< 0.001), respectively (66).

Spinal metastasectomy has had mixed outcomes. In 2021, Kato et al. published their findings on a retrospective cohort of 65 mRCC patients with spinal metastases who underwent MTX (*n* = 38, 58.5%) or iMTX (*n* = 27, 41.5%) (49). Cancer-specific survival (CSS) in the MTX group was significantly improved compared to the iMTX group (89.5%, 76.7%, and 60.3% vs. 59.3%, 42.0%, and 31.5% for 3-, 5- and 10-year CSS, respectively; *p* < 0.01). In contrast, Ptashnikov et al. reported on 100 mRCC patients with spinal metastases, of whom 39 (39%) underwent MTX and the remainder underwent non-MTX decompression and stabilization; they observed no significant difference in median OS between the MTX (median OS 22 months, 95% CI, 18–30) and non-MTX (median OS 22 months, 95% CI, 16–32; *p* = 0.075) groups (39).

Many prognostic factors in bone mRCC have been identified through multivariate analysis and include number of bone metastases, concomitant visceral metastases, local recurrence, sarcomatoid dedifferentiation, and Fuhrman grade (Table 3) (49, 50, 66, 68, 69). Kato et al. identified Eastern Cooperative Oncology Group (ECOG) performance status 3 (OS HR 51.4, 95% CI, 10.7–245.6; *p* < 0.01) and presence of concomitant liver metastasis (OS HR 89.7, 95% CI, 5.98–1344.4; *p* < 0.01) as strong prognosticators of poor OS (49). The presence of multiple spinal metastases (HR 5.7, 95% CI, 1.14–28.7; *p* = 0.03) and incomplete metastasectomy (HR 3.0, 95% CI, 1.09–8.42; *p* = 0.03) were also identified as poor prognosticators of OS. In 2019, Ruatta et al. identified the presence of concomitant visceral metastasis (OS HR 2.02, 95% CI, 1.39–2.96; *p* < 0.05) as a poor prognosticator, while low Memorial Sloan Kettering Cancer Center (MSKCC) risk group, complete bone metastasis resection, and the presence of synchronous solitary bone metastasis were all protective factors (68).

**Table 3:** Prognostic factors identified *via* multivariate analysis for metastatic renal cell carcinoma in patients with bone metastases.

Study	Year	Study Description	Patient #	Prognostic Factors	OS HR/OR (95% CI)	*p*-value
Hwang et al.	2014	Retrospective, Single-center	135	Multiple bone metastases	HR 2.5 (1.3–4.7)	0.009
				≥1 Visceral metastases	HR 3.0 (1.5–6.1)	0.003
				Local recurrence	HR 3.0 (1.4–6.7)	0.007
Du et al.	2016	Retrospective, Single-center	114	Targeted therapy	HR 0.375 (0.150–0.934)	0.036
				Resection of bone metastases	HR 0.114 (0.044–0.297)	0.000
				Bisphosphonate treatment	HR 0.419 (0.196–0.894)	0.024
				Sarcomatoid features	HR 4.117 (1.307–12.973)	0.016
				Fuhrman grade	HR 0.382 (0.172–0.849)	0.018
Huang et al.	2019	Retrospective, Single-center	106	Metachronous Metastasis	OR 0.981 (0.970–0.993)	0.001
				Elderly age	OR 1.040 (1.001–1.080)	0.042
				Concomitant visceral metastases	OR 3.883 (1.375–10.967)	0.01
				Presence of Carbonic Anhydrase- IX	OR 0.017 (0.001–0.377)	0.01
Ruatta et al.	2019	Retrospective, Single-center	300	Concomitant visceral metastases	HR 2.02 (1.39–2.96)	<0.05
				Low MSKCC risk group	HR 0.5 (0.38–0.67)	<0.05
				Radical bone metastasis resection	HR 0.68 (0.50–0.93)	0.01
				Synchronous solitary bone metastasis	HR 0.66 (0.43–0.99)	0.04
Kato et al.	2021	Retrospective, Single-center	65	Postoperative disability (ECOG PS 3)	HR 51.4 (10.7–245.6)	<0.01
				Concomitant liver metastases	HR 89.7 (5.98–1344.4)	<0.01
				Multiple spinal metastases	HR 5.7 (1.14–28.7)	0.03
				Incomplete metastasectomy	HR 3.0 (1.09–8.42)	0.03

OS, overall survival; HR, hazard ratio; OR, odds ratio; MSKCC, Memorial Sloan Kettering Cancer Center; ECOG PS, Eastern Cooperative Oncology Group performance status.

Complications of bone MTX in mRCC are common and can often require reoperation. Reported complications include wound infection, prosthetic infection, pneumonia, deep vein thrombosis, and major vessel injury (49, 50, 65, 67). Hwang et al. reported complications in 8 of 135 (5.9%) patients; 2 patients experienced superficial wound infections that resolved with antibiotics, and 6 patients had deep prosthetic infections, with one patient requiring an above-knee amputation as a result (50). They also reported that 1- and 2-year cumulative risk of revision was 4% and 8%, respectively. Langerhuizen et al. observed a 5-year reoperation probability for patients with negative surgical margins and positive surgical margins of 0.23 (95% CI, 0.15–0.44) and 0.65 (95% CI, 0.36–0.92), respectively (49, 65, 67).

The literature regarding bone MTX for mRCC has mixed results and complications can be severe or require reoperation. Patients should be selected for good prognostic factors for survival when undergoing MTX with bone metastasis such as having a good performance status, a solitary bone lesion and the ability to completely resect the lesion.

### Liver

Liver is the 3rd most common site of metastasis, occurring in 19% of mRCC cases (51). Kim et al. observed that mRCC patients with liver metastases have a poor prognosis, with a median OS of 7.4 months (32). In 2020, Beetz et al. reported on 40 mRCC patients with liver metastases who underwent MTX, 14 (35%) of whom had concomitant extrahepatic metastases, and found a significant difference in median OS between the two groups (70). Patients without extrahepatic metastases had a median OS of 47.2 months compared to a median OS of 23.4 months in those with extrahepatic metastases (*p* = 0.017). Finally, an older study by Thelen et al., published in 2007, reported on 31 patients who underwent liver MTX and demonstrated 1-, 3-, and 5-year OS of 82.2%, 54.3%, and 38.9%, respectively (71).

In 2010, Staehler et al. published a study of 88 mRCC patients, 68 (77.3%) of whom underwent MTX, and 20 (22.7%) who were offered MTX but declined (non-MTX) (26). They observed that, with baseline characteristics not being statistically different between groups, the MTX group fared much better compared to the non-MTX group, with a median OS of 142 months (95% CI, 115–169) vs. 27 months (95% CI, 16–38; *p* = 0.003), respectively. More recently in 2019, Kim et al. observed a more favorable median OS in mRCC patients with liver metastases who underwent MTX (median OS 25.97, 95% CI, 12.79–61.81) compared to their non-MTX counterparts (median OS 9.86 months, 95% CI, 8.19–12.79; *p* = 0.013) (32).

Prognostic factors for liver mRCC patients that have been identified by multivariate analysis include Fuhrman grade, synchronous versus metachronous metastasis, ECOG performance status, and resection margins, among others (Table 4) (26, 32, 70, 71). Beetz et al. demonstrated that multivisceral metastasis resection (HR 9.851, 95% CI, 2.715–35.737; *p* = 0.001) was a poor prognosticator of OS, while a longer interval from nephrectomy to hepatic MTX (HR 0.971, 95% CI, 0.956–0.987; *p* < 0.001) was favorable (70). Similarly, Kim et al. reported that patients with concomitant liver and lung metastasis had poorer OS (HR 2.62, 95% CI, 1.23–5.58; *p* = 0.0126) and PFS (HR 2.62, 95% CI, 1.07–4.58; *p* = 0.0320) (32).

**Table 4:** Prognostic factors identified *via* multivariate analysis for metastatic renal cell carcinoma in patients with liver metastases.

Study	Year	Study Description	Patient #	Prognostic Factors	Endpoint HR/OR (95% CI)	*p*-value
Thelen et al.	2007	Retrospective, Single-center	31	Resection margins	OS Not Recorded	0.005
Staehler et al.	2010	Retrospective, Single-center	88	Fuhrman grade	OS HR 2.568 (1.238–5.329)	0.011
				Initial T-stage	OS HR 3.712 (1.440–9.552)	0.007
				ECOG PS ≥1	OS HR 3.763 (1.777–7.972)	0.001
				Liver MTX	OS HR 2.230 (1.054–4.719)	0.036
Kim et al.	2019	Retrospective, Single-center	273	Concomitant Liver + Lung Metastasis	PFS HR 2.22 (1.07–4.58)	0.0320
					OS HR 2.62 (1.23–5.58)	0.0126
Beetz et al.	2020	Retrospective, Single-center	40	Multivisceral resection	OS HR 9.851 (2.715–35.737)	0.001
				Longer interval from nephrectomy to hepatic MTX	OS HR 0.971 (0.956–0.987)	<0.001

HR, hazard ratio; OR, odds ratio; OS, overall survival; PFS, progression-free survival; ECOG PS, Eastern Cooperative Oncology Group Performance Status; MTX, metastasectomy.

Complication rates in liver MTX for mRCC are relatively high, with significant morbidity and mortalities reported in the literature. Complications include wound infection, biliary leakage, hemorrhage, respiratory insufficiency, lung embolism, atrial fibrillation, pneumothorax and abscess formation (26, 70–73). Meyer et al. reported that the resection of hepatic lesions was significantly associated with higher odds of any complications (HR 2.59, 95% CI, 1.84–3.62; *p* < 0.001) (51). These findings were not true for the resection of any other metastasis sites on univariate analysis. Staehler et al. reported perioperative morbidity of 20.1% (*n* = 15), complications of which included bleeding, biliary leakage, abscess formation, pleural effusion, and paralytic ileus (26). In 2020, Beetz et al. reported a postoperative complication rate of 41.0% (*n* = 16), with 9 patients (23.1%) experiencing severe complications (≥ Clavien-Dindo grade III) (70). Perioperative mortality occurred in 2 (5%) patients, secondary to post-hepatectomy liver failure and postoperative hemorrhage, respectively (71–73). These findings demonstrate that the survival benefit of liver MTX must be weighed against the significant risk of post-operative morbidity and mortality to select the correct patient population to undergo surgical management for liver metastases in mRCC.

### Adrenal

mRCC involves the adrenal glands in 11% of cases (51). While this represents a significant patient population, few papers have evaluated outcomes following adrenal MTX. In 2001, Paul et al. conducted a retrospective analysis of 866 consecutive patients who underwent simultaneous radical nephrectomy and ipsilateral adrenalectomy for RCC (74). 27 patients (3.1%) were found to have adrenal metastases, of whom 6 had local invasion of the adrenal gland while 21 had hematogenous spread. This study did not distinguish between results for those with hematogenous spread versus those with contiguous tumor. mRCC patients with adrenal involvement were reported to have mean OS of 15.3 months, compared with 45.1 months for those mRCC patients without adrenal involvement (*p* < 0.0001).

In 2004, Siemer et al. conducted a retrospective study of 1,635 RCC patients who underwent radical nephrectomy alone (*n* = 625) or radical nephrectomy and ipsilateral adrenalectomy (*n* = 1,010) (75). Of those who underwent concurrent adrenalectomy, 56 (5.5%) were found to have metastatic disease to the ipsilateral adrenal gland. In this subgroup, patients with a solitary adrenal metastasis (*n* = 18) had better survival rates than those with multiple metastases (*n* = 36) (5-year OS 61% vs. 19.6%; *p* < 0.05). More recently, Nerli et al. prospectively followed RCC patients treated with partial or radical nephrectomy for the subsequent development of adrenal masses; in their cohort, 8 patients developed adrenal lesions and underwent laparoscopic MTX (76). Mean OS was determined to be 44.62 months, and no perioperative morbidity or mortality was observed. No data regarding outcomes for MTX vs. non-MTX patients with adrenal metastases were identified. Further studies regarding adrenal metastasis in mRCC must be done to better evaluate the impact of MTX in this setting.

### Brain

Brain metastases have been reported to occur in 3.4% of all mRCC cases (51). In 2018, Sun et al. published a study on 2,911 mRCC patients including 208 patients with brain metastases (22). From this population, multivariate analysis revealed a significant association between CSM and non-MTX patients (HR 1.61, 95% CI, 1.28–2.02; *p* < 0.001). In 2013, Naito et al. published a multi-institutional study of 534 mRCC patients, 38 (7.1%) of whom had brain metastases, and demonstrated a median OS of 11.2 months (Standard Error 6.1) versus 87.3 months (Standard Error 6.6) in the brain metastasis and non-brain metastasis groups, respectively (*p* < 0.001) (77). Furthermore, on multivariate analysis, brain metastasis at time of metastasectomy was associated with poor outcomes (HR 3.73, 95% CI, 2.03–6.86; *p* < 0.001). In 1998, Kavolius et al. conducted a retrospective study of 278 RCC patients, 11 (4.0%) of whom had solitary brain metastases, and determined the 5-year OS to be 18%. Sun et al. also evaluated survival outcomes for PSM mRCC patients with brain metastases who underwent MTX versus those who did not; 1-, 2-, and 3- year OS rates were 71.1%, 51.2% and 41.3% vs. 46.8%, 36.2% and 29%, respectively, *p* = 0.047 (22). While brain metastasis in mRCC carries a poor prognosis, metastasectomy in this patient population may confer a survival benefit for the appropriately selected patient.

### Pancreas

While RCC is the most common primary tumor to metastasize to the pancreas (78), the pancreas is an uncommon site of metastasis in mRCC. A large retrospective study conducted by Shin et al. in 2021 identified 300 mRCC patients with pancreatic metastases from a total of 3,107 mRCC patients in the NIS database and determined that compared to other mRCC patients, this population was both significantly younger at diagnosis and comprised of more females (79). They also determined that the metastasis-free duration was significantly longer in this population versus other mRCC patients (median 82.0 months, 95% CI, 31.0–141.0 vs. 33.0 months, 95% CI, 14.0–72.0; *p* < 0.001). With regards to OS, patients with pancreatic metastases fared better than mRCC patients with other metastases (median OS 38.4 months, 95% CI, 18.4–72.0 vs. 20.1 months, 95% CI, 8.7–41.1, respectively; *p* < 0.001). Finally, mRCC patients with pancreatic metastases were more likely to present with metachronous metastases, clear cell histology, and low (1–2) Fuhrman grade.

Shin et al. also reported on MTX outcomes, demonstrating an improvement in OS only for mRCC patients with metachronous pancreatic metastases (median OS 53.7 months, 95% CI, 25.3–83.4 vs. 45.1 months, 95% CI, 20.4–67.8; *p* = 0.012) (79). On multivariate analysis, pancreas MTX was a significant prognosticator overall for OS (HR 0.482, 95% CI, 0.252–0.921; *p* = 0.027). Malleo et al. in 2021 reported on 69 mRCC patients with pancreatic metastases from two institutions; 5- and 10-year recurrence rates following MTX were 53.7% and 62.7%, respectively (80). Furthermore, they found that extended pancreatectomy was associated with a 3-fold relative incidence of new recurrent disease when compared to standard pancreatectomy (adjusted subdistribution HR 3.05, 95% CI, 1.72–5.40; *p* = 0.001). In patients who do have recurrence following pancreatectomy, 5- and 10-year CSM rates were 27.1% and 35.4%, respectively. One contradictory study by Santoni et al. in 2015, which retrospectively reviewed 103 consecutive mRCC patients with pancreatic metastases, demonstrated that OS was not significantly improved in patients who underwent MTX compared to those who were treated with a tyrosine kinase inhibitor (TKI) alone (median OS 103 months, 95% CI, 75 – NR vs. 86 months, 95% CI, 80-NR; *p* = 0.201) (81).

Prognostic factors for mRCC patients with pancreatic metastases identified across studies on multivariate analysis include Heng risk group, MSKCC risk group, and T-stage (Table 5) (79, 81). Shin et al. identified stage T4 disease as a strong, poor prognostic factor for patients with pancreatic metastasis (OS HR 27.380, 95% CI, 3.166–236.773; *p* = 0.003) (79). Likewise, Shin et al. determined that intermediate Heng risk group was also associated with worse OS (HR 2.614, 95% CI, 1.386–4.930; *p* = 0.003).

**Table 5:** Prognostic factors identified *via* multivariate analysis for metastatic renal cell carcinoma in patients with pancreas metastases.

Study	Year	Study Description	Patient #	Prognostic Factors	OS HR/OR (95% CI)	*p*-value
Santoni et al.	2015	Retrospective, Multi-center	103	MSKCC risk group	HR 5.14 (0.98–27.0)	0.04
Shin et al.	2021	Retrospective, Multi-center	300	Intermediate Heng risk group	HR 2.614 (1.386–4.930)	0.003
				T-stage 4	HR 27.380 (3.166–236.773)	0.003
				Pancreatic MTX	HR 0.482 (0.252–0.921)	0.027

OS, overall survival; HR, hazard ratio; OR, odds ratio; MSKCC, Memorial Sloan Kettering Cancer Center; MTX, metastasectomy.

Complications in pancreatic MTX for mRCC are common, including pancreatic fistula, delayed gastric emptying, post-pancreatectomy hemorrhage, intestinal bleeding, acute respiratory distress syndrome and wound infections (80, 82, 83). Malleo et al. reported a morbidity rate of 34.8% (*n* = 24) (80) and mortality rate was 2.9% (*n* = 2) in the perioperative period (80, 82, 83). Pancreatic MTX has been associated with improved survival, but both recurrence and perioperative morbidity are common.

## Other metastatic sites

Thyroid metastasis is a rare event in mRCC, and data for this patient population are limited. Two studies on thyroid MTX for mRCC were identified. In 2015, Beutner et al. conducted a retrospective, single-center study for 34 patients and performed a systematic review including 32 studies with 285 patients (84). In their retrospective analysis, median time to primary metastasis from primary RCC resection was 6.5 years and median survival after primary metastasis was 4.7 years (95% CI, 1.8–7.6). On systematic review, median time to metastasis, excluding synchronous cases, was 8.8 years (95% CI, 7.5–10.1) and median actuarial survival after thyroid metastasis was 3.4 years (95% CI, 2.2–4.6). Survival was not significantly improved in the MTX versus iMTX groups. In 2008, Iesalnieks et al. conducted a multi-institutional, retrospective analysis of 45 mRCC patients who underwent thyroid MTX. 5-year OS was 51% following MTX (85). On multivariate analysis, elderly age (≥70 years old) was a significant prognosticator for poor survival. It was also observed that of 45 patients with thyroid metastases, 14 (31%) developed pancreatic metastases during the disease course.

Bladder metastasis is also a very uncommon event in mRCC. In 1981, Saitoh reviewed 1,451 autopsy results from patients with RCC and reported the rate of bladder metastasis to be 2% (*n* = 23), with only 1 patient having a solitary bladder metastasis (86). In 2015, Matsumoto et al. published a systematic review and analysis of mRCC patients with bladder metastases (87). Of the 65 patients identified, 58 patients had data available regarding metastases. Median time from diagnosis of RCC to metachronous bladder metastasis was 33 months. 36 patients (62%) had bladder metastases only, while 22 (38%) had additional sites of metastasis. The 2-year CSS in patients with solitary bladder metastasis was 71.1% versus 25.8% in those with additional metastasis sites (*p* = 0.007). Multivariate analysis revealed that patients with bladder metastasis within 1 year from RCC diagnosis (CSS HR 3.25, 95% CI, 1.05–10.1; *p* = 0.042) and patients with additional metastases (CSS HR 3.88, 95% CI, 1.42–10.6; *p* = 0.008) had poorer outcomes.

## Risk stratification

Historically, two prognostic tools have been utilized in the risk stratification of mRCC patients, the MSKCC/Motzer risk grouping and the IMDC/Heng risk model. Both models have been externally validated and are effective in predicting outcomes for mRCC patients (88–91). Multiple studies that have already been discussed in this text have demonstrated beneficial outcomes for mRCC patients with favorable-risk grouping in the MSKCC and IMDC models who undergo MTX, while patients with poor-risk stratification may fail to benefit from MTX (33, 68, 79, 81). More recently, other models have been created, including the Munich score by Meimarakis et al. and a nomogram developed by Wu et al. based on data from the Surveillance, Epidemiology, and End Results (SEER) database (24, 46).

In 2011, Meimarakis et al. utilized data from 175 consecutive mRCC patients who underwent pulmonary MTX to create a novel prognostic score that would predict survival (46). Their predictive scoring system, called the Munich score, utilized six factors that were identified *via* multivariate analysis: (1) pleural infiltration, (2) synchronous manifestation of primary RCC and pulmonary metastases, (3) nodal status of primary tumor, (4) metastasis size >3 cm, (5) mediastinal and/or hilar lymph node metastases, and (6) completeness of metastasectomy. These criteria were the result of parameters first established by the International Registry of Lung Metastases and Hoffman et al., upon which Meimarakis et al. then expanded (47, 92). Patients in the low-risk, intermediate-risk, and high-risk groups had complete resection (R0) and no risk factors, R0 and ≥1 risk factor, and incomplete resection (R1 or R2), respectively. The Munich score system demonstrated significant differences in median OS between the low, intermediate, and high-risk groups (90.1 months, 95% CI, 53.3–127.0 vs. 31.4 months, 95% CI, 22.1–40.7 vs. 14.2 months, 95% CI, 11.5–17.0, respectively).

In 2020, Wu et al. utilized data from 2,911 mRCC patients in the SEER database and created a novel nomogram to predict survival (24). Similar to the Munich score, this nomogram utilized prognostic factors identified on multivariate analysis to assign patients to low, intermediate, and high-risk groups. 3-year CSM was 35.6% in the low-risk, 59.0% in the intermediate-risk, and 80.4% in the high-risk group (*p* < 0.001).

## Histologic & molecular subtypes

Histologic subtypes can serve as significant prognosticators for clinical outcomes in mRCC patients. Generally, clear-cell histology seems to confer a strong benefit for survival (21, 25, 41, 65, 93). In 2007, Lin et al. reported 1- and 5- year OS for 248 clear-cell mRCC patients and 47 non-clear-cell mRCC patients (51.0% and 12.0% vs. 25.0% and 0%, respectively; *p* < 0.0001), demonstrating a significant OS benefit for those with clear-cell histology (65). Likewise, in 2019, Kim et al. published a study of 156 non-clear-cell mRCC patients who were treated with targeted therapy and reported superior first-line PFS (median 8.0 vs. 5.0 months, *p* = 0.0008), total PFS (median 12.0 vs. 6.0 months, *p* = 0.0002), and CSS (median 31.0 vs. 24.0 months, *p* = 0.0272) in mRCC patients with clear-cell versus those with non-clear-cell histology (41). On the contrary, one study in 2019, which reported data on 273 mRCC patients, observed that on both univariate (HR 0.53, 95% CI, 0.35–0.79; *p* = 0.0021) and multivariate (HR 0.61, 95% CI, 0.40–0.93; *p* = 0.0205) analysis, non-clear-cell histology conferred a slight benefit for PFS (32).

In 2015, Beuselinck et al. utilized unsupervised transcriptome analysis of 53 mRCC patients to identify 4 clear-cell RCC subtypes (ccrcc1 to 4), that differ in their mRNA expression, methylation status, mutation profile, cytogenic anomalies, and immune infiltrate (94). When grouped, ccrcc1 and ccrcc4 tumors had a lower response rate to first-line sunitinib therapy (*p* = 0.005) and a shorter PFS and OS than ccrcc2 and ccrcc3 tumors (*p* = 0.001 and 0.0003, respectively). A follow-up study conducted by Verbiest et al. in 2018 examined these 4 subtypes and outcomes following MTX in 43 mRCC patients (95). Median DFS after MTX was 23 months for ccrcc2 and ccrcc3 tumors versus 9 months for ccrcc1 and ccrcc4 tumors (HR 2.56; *p* = 0.011). Likewise, OS was significantly better in the ccrcc2 and ccrcc3 group (Median OS 127 months vs. 50 months, HR = 2.54; *p* = 0.011).

Sarcomatoid features on histology have also been widely reported as a poor prognosticator in mRCC (34, 66, 96). In 2020, Holz et al. reported on 138 mRCC patients who underwent MTX, and observed a significant impact on OS for patients with sarcomatoid dedifferentiation in the primary tumor (HR 4.52, 95% CI, 1.15–17.69; *p* = 0.03) (62). In 2014, Beuselinck et al. reported on 117 mRCC patients who underwent MTX & TKI therapy, and observed that OS and PFS were very poor in those patients with sarcomatoid dedifferentiation in ≥25% of the primary tumor histology compared to those with <25% or no sarcomatoid histology (97). Multivariate analysis for OS and PFS revealed HR 2.885 (95% CI, 1.380–6.028; *p* < 0.0001) and HR 4.446 (95% CI, 2.084–9.486; *p* = 0.005), respectively. Similarly, in 2018 Korenbaum et al. reported on 47 mRCC patients and, utilizing a cutoff of >30% sarcomatoid dedifferentiation, found a significant impact on OS *via* univariate analysis (HR 2.9, 95% CI, 1.267–6.643; *p* = 0.02) (98).

Finally, in 2020, Kim et al. analyzed concordance in the expression of 20 different tissue markers utilizing 162 metastasectomy tissue samples from 66 mRCC patients (99). Among these markers, BRCA1-associated protein-1 (BAP1), prostate-specific membrane antigen (PSMA), vascular endothelial growth factor receptor 3 (VEGFR3), platelet-derived growth factor receptor *α* (PDGFR*α*), and phosphorylated S6 (pS6) demonstrated a high concordance ratio (>0.7), regardless of different metastatic tissues and different metastatic lesions within the tumor. BAP1 loss was observed in 99.0% of all metastasectomy tissue samples of the same organ, 96.4% of different metastatic organs in the same patient, and 100% of recurrent tissues from the same organ. Another study by da Costa et al. in 2019 identified BAP1 loss as a poor prognosticator for OS. 5-year OS rates for patients with BAP1 positive and BAP1 negative tissue were 53.2% and 35.1%, respectively (*p* = 0.004) (100). Likewise, BAP1 loss was associated with higher risk of death (HR 1.913; *p* = 0.041) and disease progression (HR 1.656, *p* = 0.021).

## Guidelines for metastasectomy in mRCC

The American Urological Association (AUA) provides an expert opinion, stating that “surgical resection or ablative therapies should be considered in select patients with isolated or oligo-metastatic disease” (101). The AUA further explains that patients should have good performance status, and that the consideration for surgical intervention should be considered in a multidisciplinary discussion. The European Association of Urology (EAU) also recommends that surgery should be discussed within the context of a multidisciplinary team (102). The EAU guidelines indicate that metastasectomy is an appropriate local treatment for most metastatic sites, aside from brain and, in some cases, bone metastases. Furthermore, for patients with brain and bone metastases, stereotactic radiotherapy can provide significant relief from local symptoms. The National Comprehensive Cancer Network (NCCN) states that those with a potentially resectable primary RCC with oligometastatic sites are candidates for nephrectomy and management of metastases by surgical metastasectomy or with ablative techniques for those who are not candidates for metastasectomy (103). The NCCN further states that candidates include patients who develop oligometastases after a prolonged disease-free interval from nephrectomy. The American Society of Clinical Oncology (ASCO) states definitive metastasis-directed therapy may be offered for low-volume mRCC including surgical resection, ablative therapy or radiotherapy (104). TKIs after complete MTX is not recommended. For brain metastases in clear cell RCC, ASCO recommends local therapy with radiation and/or surgery (104).

## Indications for metastasectomy in clinical practice

Multiple studies have demonstrated that metastasectomy in the proper clinical setting can improve patient outcomes. Multiple prognostic factors identified through retrospective studies demonstrate common variables that should be considered in the patient selection process. These favorable factors include if complete resection of metastatic disease is feasible (46, 49, 56–59, 68), solitary or oligometastatic disease (36, 38, 46, 55, 57), metastatic disease isolated to a single organ and those lacking lymph node involvement of the primary tumor (32, 36, 46, 49, 50, 52, 56, 58, 60, 61, 68–70), younger patients, especially under 60 years old (53, 54, 60, 69), clear-cell histology (21, 25, 41, 65, 93), and lung metastases (22).

Additionally, use of the risk stratification tools has been shown to be an effective way to determine the prognosis of patients with mRCC, and this data is then extrapolated to determine which patients may be more fit for surgery. Multiple studies have demonstrated that patients with favorable-risk grouping in the MSKCC and IMDC models are more likely to benefit from metastasectomy, while patients with poor-risk stratification may not benefit from surgery (33, 68, 79, 81). The risk stratification tools in combination with various prognostic factors should be taken together when deciding if a patient would likely benefit from a metastasectomy.

## Patient quality of life after metastasectomy

While the survival benefits associated with metastasectomy have been well-documented in the literature, patients who undergo metastasectomy must also consider their quality of life post-operatively and consider how alternative therapies may shape their futures. In those patients who are good candidates for complete surgical resection of metastases, CN and MTX can achieve a residual tumor-free status. This therapeutic approach confers a significant advantage to patients who may avoid toxicities associated with systemic therapies without compromising survival (27, 105, 106). Furthermore, for patients who undergo MTX but have recurrence or progression of disease, targeted therapies may be resumed. On the contrary, patients who undergo MTX must be aware of the morbidity and mortality associated with surgery, which is dependent upon factors such as metastatic site or number of metastases. In some cases, such as those patients with mRCC and liver involvement, complication rates associated with MTX have been reported to be as high as 41.0%, with 23.3% of patients experiencing severe complications (70). Clinicians and patients must discuss the various therapeutic options available and determine patient-specific goals of care when selecting the appropriate therapy in order to ensure patients may achieve an optimal quality of life.

## Incorporation of systemic therapy with metastasectomy

In addition to examining the effects of MTX on mRCC patient populations, various retrospective studies have also reported on patient outcomes for those treated with MTX and systemic therapies versus those who receive MTX alone, albeit with relatively small sample sizes.

In 1992, Pogrebniak et al. reported on 23 mRCC patients who underwent pulmonary MTX (*n* = 15) or iMTX (*n* = 8). 18 (78.3%) patients had received neoadjuvant interleukin-2 (IL-2) immunotherapy prior to MTX, while the remainder did not (48). Of the patients who underwent MTX, no difference was observed in post-operative OS between those who received immunotherapy (mean OS 42.4 months) versus those who received MTX alone (mean OS 32.2 months, *p* = 0.73).

More recently in the era of targeted therapy, Park et al. reported on 53 mRCC patients who underwent targeted therapy and MTX, with a subgroup (*n* = 19, 35.9%) receiving post-operative targeted therapy (107). Differences in clinical outcomes were measured between those who stopped targeted therapy after surgery versus those who continued, and it was observed that risk for recurrence was decreased in those who continued immunotherapy after MTX (HR 0.418, 95% CI, 0.118–0.859; *p* = 0.017). However, when comparing the groups for CSS, no significant differences were observed (HR 0.640, 95% CI, 0.258–2.093; *p* = 0.714).

In 2020, Verbiest et al. reported on 113 mRCC patients who underwent MTX, with 59 patients (52.2%) starting systemic therapy in the post-operative period (108). 41 patients in this group received first line sunitinib or pazopanib, and the reported PFS and CSS were 18 months and 35 months, respectively, with a response rate of 50%. No comparative analyses between these populations were conducted. However, they reported that for the 59 patients who began systemic therapy in the follow-up period, the median time to start systemic therapy was 32 months after relapse, which indicates an indolent course of disease for patients who previously underwent MTX.

In addition to retrospective studies, RCTs have also investigated the value of systemic therapy use in conjunction with metastasectomy. Procopio et al. reported on Phase 2 results of the RESORT study, which compared outcomes for patients treated with MTX and adjuvant sorafenib versus those who underwent MTX alone (109). Overall, 69 patients were randomized to receive MTX & sorafenib (*n* = 33) or MTX alone (*n* = 36). No significant differences were observed between the two groups (median recurrence free survival [RFS] 21 months, 95% CI, 11 – NR vs. 37 months, 95% CI, 20 – NR; *p* = 0.404). In addition, patients in the sorafenib arm experienced more adverse events (84% vs. 31%) and more high-grade (≥ grade 3) adverse events (31% vs. 3%). An update on this study published in 2021 by Mennitto et al. further demonstrated no RFS benefit with sorafenib (HR 1.35, 95% CI, 0.72–2.54; *p* = 0.342) (110).

In 2019, Rausch et al. reported on Phase 1/2 results of an RCT which examined the use of an adjuvant multi-peptide vaccine (UroRCC) after metastasectomy (*n* = 19) in comparison to metastasectomy alone (*n* = 44) (111). This study demonstrated a significant survival benefit for those receiving UroRCC (median OS NR, mean OS 112.6 months, 95% CI, 92.1–133.1) versus those who underwent MTX alone (median OS 57.96, 95% CI, 37.2–63.1; *p* = 0.015). After adjusting for MSKCC risk groups, metastasis sites, and age, receipt of UroRCC remained an independent prognostic factor for OS (HR 0.19, 95% CI, 0.05–0.69; *p* = 0.012). Furthermore, the UroRCC vaccine was observed to be well-tolerated, and the majority of adverse events were Grade 1, with one patient experiencing reactivation of sarcoidosis and another experiencing skin necrosis at the injection site. Overall, the UroRCC vaccine has shown promising results with limited adverse effects.

PROSPER RCC (NCT03055013) is another ongoing clinical trial which is investigating recurrence-free survival in high-risk RCC patients treated with perioperative nivolumab (112). Patients are assigned to receive both neoadjuvant and adjuvant nivolumab with partial/radical nephrectomy versus surgery alone. This study allows for the inclusion of patients with oligometastatic disease, given that they are planned to undergo local treatment for metastases within 12 weeks of nephrectomy and that the metastatic site is not located in liver, bone, or brain. This trial is active and not recruiting.

Finally, KEYNOTE-564 (NCT03142334) published results in 2021, reporting on the phase 3 results for high-risk RCC patients who received adjuvant pembrolizumab (*n* = 496) or placebo (*n* = 498) (113). This study also included patients who were treated with or without metastasectomy. Pembrolizumab therapy had a positive impact on DFS at 24 months (77.3% vs. 68.1%; HR for recurrence or death 0.68, 95% CI, 0.53–0.87; *p* = 0.002). Overall, 386 patients (79.1%) who received pembrolizumab and 265 (53.4%) who received placebo had at least one adverse event. However, metastasectomy-specific outcomes were not included in this report.

## Alternatives to metastasectomy for MRCC

### Ablative therapies

There are alternative treatment options available for mRCC patients which are less invasive in comparison to surgical intervention, such as radiofrequency ablation (RFA), stereotactic ablative radiotherapy (SABR), and cryoablation (CA) (114). RFA is performed by either percutaneously or laparoscopically inserting a probe into the tumor to administer radiofrequency waves that cause tissue destruction (114). CA involves inserting a cryoprobe into a tumor which then rapidly removes heat from the tissue to cause tissue necrosis (115). SABR is a more potent form of radiofrequency treatment which delivers high dose radiation that is concentrated at the tumor site, with limited radiation reaching surrounding tissue (116).

RFA and CA have been used as treatment options to target metastatic lesions for patients with mRCC. In 2014, Welch et al. reviewed 61 patients with mRCC who underwent percutaneously-guided RFA and CA of metastases, with the majority of metastatic sites located in the liver (21%) and adrenal gland (17%) (117). The estimated RFS rates at 1, 2, and 3 years after ablation were 94%, 94% and 83%, respectively (117). They also found estimated OS rates at 1, 2, and 3 years after ablation to be 87% (95% CI, 79–97), 83% (95% CI, 73–94), and 76% (95% CI, 63–90), respectively (117).

Several studies have reported on RFA for treating lung metastases in patients with mRCC. In 2019, Gonnet et al. conducted a retrospective study on 53 mRCC patients with 100 total lung metastases treated by RFA and found a 5-year OS of 62% (95% CI, 44–75) after a median follow-up of 61 months (IQR 34–90) (118). Three major complications were observed, and 25% of patients who experienced lung recurrence after RFA were treated with additional RFA (118). This study observed no procedural-related deaths and demonstrated low morbidity risk with repeated procedures (118). A subgroup analysis by da Baere et al. in 2015 of 566 patients treated with RFA for mRCC with metastases to the lungs had low rates of local treatment failure (7.4% and 25.1% at 1 and 3 years, respectively) with an OS rate of 73.5% at 3 years (119).

In addition to RFA and CA, studies have evaluated the efficacy of SABR in treating mRCC. A systematic review by Cheung et al. in 2014 demonstrated the efficacy and palliative effects of SABR for mRCC patients (120). Furthermore, the study suggested that the addition of immunotherapy with SABR enhanced the abscopal effect of radiotherapy (120). In 2012, A phase I trial conducted by Seung et al. included 12 patients with either metastatic melanoma or mRCC who were treated with a combination of SABR and IL-2. All patients were observed to have a partial or CR to treatment with a response rate of 67%, compared with a response rate of 12% with IL-2 alone (121). In 2006, Wersail et al. demonstrated tumor regression after treatment with SABR of either the primary tumor or other metastatic lesion in 4 out of 28 mRCC patients who had previously non-irradiated metastases (122). In 2019, Zaorsky et al. performed a meta-analysis of 28 studies with a combined total of 1,602 patients with oligo-metastatic RCC treated with SABR. Reported 1-year OS rates were 86.8% and 49.7% for extracranial and intracranial disease, respectively (123). In 2015, Kothari et al. also performed a systematic review and reported on outcomes of SABR for cranial and extracranial mRCC, demonstrating that OS ranged from 6.7 to 25.6 months, with median OS ranging from 11.7 to 22 months (124). These studies show ablative therapy may be considered as a less invasive alternative for the treatment of mRCC.

### Systemic therapy alone

The variety of Food and Drug Administration (FDA)-approved systemic therapies for mRCC are reviewed in Table 1. While systemic therapies have continued to evolve with the advent of immunotherapy, CR for systemic options continue to be low for mRCC. A meta-analysis of phase II–III randomized clinical trials compared differences in the incidence of CR between antiangiogenic agents (AAs) versus non-AAs as the standard comparator arm (11). The AAs administered in the experimental arm were sunitinib, sorafenib, pazopanib, and bevacizumab. The incidence of CR in patients treated with AAs was 2.0% (95% CI, 1.2–2.8) compared to 1.4% (95% CI, 0.7–2.1) in the non-AA control arm. Specific AAs were also compared to each other, and the incidence of CR was 2.5% (95% CI, 1.2–3.8) in the bevacizumab group and 1.6% (95% CI, 0.1–2.5) in the TKIs group.

The curative potential for cytokine therapies such as high dose IL-2 has been explored in populations of patients with metastatic disease. An RCT of 156 patients by Yang et al. in 2008 compared response rates of patients treated with high dose IL-2 versus those treated with low dose IL-2 in the control arm (7). There was a higher response proportion of 21% in the high dose IL-2 cohort compared to 13% in the control arm. Within the high dose IL-2 experimental arm, CR was 7% and PR was 14%, while CR was 4% and PR was 8% within the control arm (7). Another RCT by McDermott et al. in 2005 with 192 patients compared differences in outcomes between high dose IL-2 and outpatient IL-2 with IFN-α in the control arm (6). The response rate of high dose IL-2 was 23.3% compared to 9.9% for patients who received IL-2 and IFN-α (6). Recent advancements, however, have supplanted the use of IL-2 with targeted therapy such as TKIs (125).

The efficacy of TKIs, mammalian target of rapamycin (mTOR) inhibitors, and VEGFRs has been well-explored through RCTs (126). A phase 3 RCT by Motzer et al. in 2007 enrolled 750 patients and compared response rates for those treated with sunitinib versus IFN-α (127). PFS was significantly longer in the sunitinib group at 11 months versus 5 months for the IFN-α group. The objective response rate (ORR) was also higher for the sunitinib cohort (31% vs. 6%, respectively) (127). In 2009, another phase 3 RCT by Escudier et al. assessed the final efficacy and safety of sorafenib compared to placebo in advanced mRCC in 900 patients. The final OS after censoring post-cross-over placebo survival data was significantly longer in patients receiving sorafenib versus the placebo group (17.8 months vs. 14.3 months, HR 0.78; *p* = 0.029) (128). A 2020 phase 3 RCT by Rini et al. studied 350 patients with mRCC treated with at least two prior systemic treatments. They were assigned to receive either tivozanib (*n* = 175) or sorafenib (*n* = 175). Median PFS was significantly longer with tivozanib (5.6 months, 95% CI, 5.29–7.33) than with sorafenib (3.9 months, 95% CI, 3.71–5.55, HR 0.73, 95% CI, 0.56–0.94; *p* = 0.016) (129).

Motzer et al. conducted another phase 3 RCT in 2015 with 416 mRCC patients randomized to receive either everolimus or a placebo (4). The median PFS for patients in the everolimus group was 4.9 months versus 1.9 months in the control group (HR 0.33; *p* < 0.001) (4). The median OS of the everolimus group was 14.8 months versus 14.4 months in the control group (HR 0.87; *p* = 0.162) (4). In 2007, Hudes et al. conducted a phase 3 RCT with 626 patients who were assigned to receive either temsirolimus, IFN-α, or temsirolimus plus IFN- *α*, with the temsirolimus group demonstrating significantly improved OS (HR 0.73; 95% CI, 0.58–0.92; *p* = 0.008) and PFS (*p* < 0.001) than the IFN-α group (5).

More recently, immune checkpoint inhibitors have been developed and utilized as either monotherapy or combination therapy (130). In 2019, Rini et al. conducted a phase 3 RCT of 861 patients assigned to receive either pembrolizumab and axitinib or sunitinib alone. The ORR was 59.3% (95% CI, 54.5–63.9) in the pembrolizumab-axitinib group versus 35.7% (95% CI, 31.1–40.4) in the sunitinib group (*p* < 0.001) (131). Nivolumab plus cabozantinib has also emerged as first-line therapy for mRCC after evidence from the 2021 phase 3 RCT by Choueiri et al. In this trial, patients were randomized to receive either nivolumab-cabozantinib (*n* = 323) or sunitinib alone (*n* = 328) (12). There was a significant survival advantage conferred in the nivolumab-cabozantinib group, with an ORR of 59.3% (95% CI, 54.5–63.9) vs. 35.7% (95% CI, 31.1–40.4) in the sunitinib control group.

A 2020 phase 2 RCT by Chung-Han Lee et al. of 104 mRCC patients who progressed on or after treatment with ICI were given lenvatinib and pembolizumab (132). After 12 weeks of therapy, they reported an ORR of 51%, median PFS of 11.7 months, and duration of response of 9.9 months (132). A phase 3 RCT of 861 patients conducted by Powles et al. in 2020 assigned patients to receive either pembrolizumab plus axitinib or sunitinib monotherapy (14). It was found that patients who received pembrolizumab and axitinib showed clinical benefit with improved OS (HR 0.53, 95% CI, 0.38–0.74) and improved PFS (HR 0.69, 95% CI, 0.57–0.84) compared to patients receiving sunitinib monotherapy. Moreover, the ORR was higher in the pembrolizumab plus axitinib group at 59.3% vs. 35.7% in the sunitinib group. Another phase 3 RCT of 651 patients performed by Choueiri et al. in 2021 studied the efficacy of nivolumab plus cabozantinib therapy compared to sunitinib (12). After a median follow-up of 18.1 months, the median PFS was 16.6 months (95% CI, 12.5–24.9) in the nivolumab plus cabozantinib group and 8.3 months (95% CI, 7.0–9.7) in the sunitinib group (HR for disease progression or death, 0.51; 95% CI, 0.41–0.64; *p* < 0.001). The probability of OS at 12 months was also higher in the nivolumab plus cabozantinib group at 85.7% (95% CI, 81.3–89.1) vs. 75.6% (95% CI, 70.5–80.0) in the sunitinib group (HR for death 0.60; 98.89% CI, 0.40–0.89; *p* = 0.001). Motzer at al. demonstrated in a phase 3 RCT in 2018 with 1096 patients that nivolumab plus ipilimumab had a superior 18-month OS compared to sunitinib (75% vs. 60%, respectively) (HR for death 0.63, *p* < 0.001). Additionally, the ORR was 42% in the nivolumab plus ipilimumab group compared to 27% in the sunitinib group (*p* < 0.001). Another phase 3 RCT by Motzer et. al in 2021 compared the efficacy of lenvatinib plus pembrolizumab, lenvatinib plus everolimus, or sunitinib alone in 1069 patients (133). OS was longer with lenvatinib plus pembrolizumab than with sunitinib (HR 0.66, 95% CI, 0.49–0.88; *p* = 0.005) but not longer with lenvatinib plus everolimus than with sunitinib (HR 1.15, 95% CI, 0.88–1.50; *p* = 0.30) with a median follow up time of 26.6 months.

Choueiri et. al conducted a phase III RCT of 886 patients in 2020 to assess the effectiveness of first-line avelumab plus axitinib therapy in comparison to sunitinib alone in the treatment of advanced RCC (125, 134, 135) Median PFS was 13.3 months (95% CI, 11.1–15.3; one-sided *p* < 0.0001) in the avelumab plus axitinib group versus 8.0 months (95% CI, 6.7–9.8) in the sunitinib group. Additionally, a phase 3 RCT by Rini et. al in 2019 compared survival of 915 patients who received either atezolizumab plus bevacizumab or sunitinib alone (136). Within the PD-L1 positive population, the median PFS was significantly better in the atezolizumab plus bevacizumab group at 11.2 months in comparison to 7.7 months in the sunitinib group (95% CI, 0.57–0.96; *p* = 0.0217). Final overall survival analysis of this trial in 2022 showed similar median OS in the intention-to-treat population at 36.1 months and the sunitinib group at 35.3 months (137). These findings demonstrate the importance of considering rapidly evolving systemic therapy alone as first-line treatment for mRCC.

## Active surveillance

Studies have also examined the role and safety of active surveillance for patients with asymptomatic and slowly progressing mRCC in an effort to avoid possible toxicities from systemic medications. In 2013 Matsubara et al. performed a retrospective analysis of 29 patients with intermediate- or favorable-risk mRCC who deferred treatment. They found that median PFS was 26.1 months, and the 4-year OS rate was 83.8% (138). Another retrospective study by Park et al. in 2014 identified 58 patients who electively deferred systemic therapy with a median time of 12.4 months (95% CI, 8.4–16.5 months) on active surveillance. 30 patients eventually underwent treatment with systemic therapy. The median follow-up time was 31 months, during which there were 15 deaths. Median OS was not reached, but the estimated median OS was 91.1 months (139). In 2012, Fisher et al. performed a retrospective study of 62 patients in the United Kingdom and examined elective deferment of first-line systemic treatment on patients with asymptomatic or slow progression of mRCC. Patients were observed for an average of 18.7 months until starting first-line therapy, after which median OS was 25.2 months (140). Within this cohort undergoing active surveillance, it was concluded that median PFS and OS times were comparable to those observed in the phase III and expanded access trials of sunitinib (127, 140, 141).

Harrison et al. in 2021 performed a prospective study, comparing mRCC patients who were initially treated with active surveillance (*n* = 143) versus systemic therapy (*n* = 305). In their study cohort, 56% of patients had a prior nephrectomy (142). Interestingly, 32% of patients in the active surveillance cohort had no evidence of disease present despite having radiologic or pathologic evidence of disease at the time of enrollment. The study demonstrated that 50% of patients in the active surveillance cohort did not receive immunotherapy after a median follow up of 42 months from time of metastatic diagnosis. Median OS of those on active surveillance was not reached versus 30 months for those on systemic therapy. While the difference in survival of the active surveillance group may possibly be related to a difference in baseline characteristics such as better ECOG performance status, improved IMDC risk profile, and with fewer metastatic sites, the authors demonstrate that active surveillance can be a safe and appropriate alternative to systemic therapy in well-selected patients.

A prospective phase II RCT by Rini et al. in 2016 enrolled 52 patients with treatment-naïve, asymptomatic mRCC to undergo active surveillance with periodic radiographic assessment (143). The decision to start systemic therapy was made at the discretion of the physician and patient, but surveillance was not required to be discontinued due to RECIST-defined progression. 37 patients eventually started systemic therapy. Median time to progression was 9.4 months (95% CI, 7.4–13.5), and the median time to initiation of systemic therapy was 14.9 months (95% CI, 10.6–25.0). The authors note that PFS was similar to that of IL-2 and sunitinib, and that active surveillance may allow subsets of mRCC patients to avoid toxicity and side effects caused by systemic therapy without compromising the benefit of therapy once initiated. These studies underscore the role of active surveillance for slow-growing mRCC in selected patients to avoid treatment-related toxicity and optimize quality of life.

## Clinical trials and clinical research

There is a lack of studies including patients who are undergoing metastasectomy for mRCC. Thirteen studies from www.clinicaltrials.gov were identified which include patients that could receive a metastasectomy in the setting of metastatic renal cell carcinoma. Two were terminated due to a lack of accrual or funding (NCT02595918 and NCT01441765). Ongoing and completed studies are shown in Table 6. One retrospective study (NCT03670992) reached completion while the other ten studies are ongoing. Five studies (NCT02210117, NCT04370509, NCT03473730, NCT03142334 [KEYNOTE-564], and NCT03055013 [PROSPER RCC]) do not require metastasectomy for every patient. For instance, NCT02210117 looks at the safety and tolerability of neoadjuvant nivolumab versus nivolumab/bevacizumab versus nivolumab/ipilimumab; however, surgical therapy may include nephrectomy, metastasectomy or biopsy after systemic therapy (144). Metastasectomy without systemic therapy was evaluated in three studies (NCT00918775, NCT03670992, NCT04245410 [PANMEKID]), with only 1 reporting results. NCT03670992 was a retrospective review of patients with pancreatic metastasis that underwent pancreatic resection without adjuvant chemotherapy; 3-, 5- and 10-year OS rates were 88.5%, 76,9% and 50%, respectively (82). Two studies (NCT02210117 and NCT04370509) are investigating the use of neoadjuvant systemic therapy followed by surgical therapy for mRCC. Two studies (NCT03473730 and NCT03055013 [PROSPER RCC]) are assessing both neoadjuvant and adjuvant systemic therapy and allow for metastasectomy. Four other studies examining outcomes of adjuvant systemic therapy allow for metastasectomy (NCT01444807 [RESORT Trial], NCT01216371 [SMAT], NCT01575548 [ECOG-E2810], and NCT03142334 [KEYNOTE-564]). NCT01444807 and NCT01575548 reported no improvement of RFS with the use of adjuvant therapy after metastasectomy (Table 6) (109, 145).

**Table 6:** Completed and ongoing clinical trials including patients undergoing metastasectomy for metastatic renal cell carcinoma.

Therapy Type	NCT Number	Study Title	Study Phase	Status	Intervention	Patient #	Primary Outcome	Results
Metastasectomy Alone	NCT00918775	Follow-up After Metastasectomy in Patients With Kidney Cancer	Phase 2	Active, Not recruiting	Follow up and evaluation during and after metastasectomy every 6 months up to 5 years	86	Progression- free/relapse free survival	None Reported
Metastasectomy Alone	NCT03670992	Surgical Treatment of Pancreatic RCC Metastases	Retrospective	Completed	Surgical removal of metastases at pancreas and/or other distal sites	26	3-, 5- and 10- year survival	3-Year OS: 88.5%
								5-Year OS: 76.9%
								10-Year OS: 50.0%
Metastasectomy Alone	NCT04245410	Surgical Treatment of Pancreatic Metastases From Renal Cell Carcinoma (PANMEKID)	Retrospective	Recruiting	Surgical treatment of pancreatic metastases	100	Overall survival	None Reported
Neoadjuvant therapy + Nephrectomy or Metastasectomy	NCT04370509	Pembrolizumab With or Without Axitinib for Treatment of Locally Advanced or Metastatic Clear Cell Kidney Cancer in Patients Undergoing Surgery	Phase 2	Recruiting	Pembrolizumab alone vs. pembrolizumab + Axitinib, Followed by cytoreductive nephrectomy or metastasectomy	84	Proportion with ≥2-fold increase in the number of tumor-infiltrating immune cells	None Reported
Neoadjuvant therapy + Nephrectomy, Metastasectomy or Biopsy	NCT02210117	Nivolumab With or Without Bevacizumab or Ipilimumab Before Surgery in Treating Patients With Metastatic Kidney Cancer That Can Be Removed by Surgery	Early Phase 1	Active, Not recruiting	Nivolumab alone vs. Nivolumab with bevacizumab or ipilimumab, Followed by nephrectomy, metastasectomy or biopsy	105	Safety and tolerability	Grade ≥3 toxicity: Nivolumab 38%
								Nivolumab + Bevacizumab 42%
								Nivolumab + Ipilimumab 47%
								1-Year OS: Nivolumab 86%
								Nivolumab + Bevacizumab 73%
								Nivolumab + Ipilimumab 83%
Neoadjuvant therapy + Nephrectomy, Metastasectomy or Biopsy + Adjuvant therapy	NCT03473730	Daratumumab in Treating Patients With Muscle Invasive Bladder Cancer or Metastatic Kidney Cancer	Early Phase 1	Active, Not recruiting	Daratumumab followed by biopsy, nephrectomy or metastasectomy. Restart daratumumab post-procedure	17	Incidence of adverse events	None Reported
Neoadjuvant therapy + Nephrectomy, Metastasectomy or Biopsy + Adjuvant therapy	NCT03055013	Nivolumab in Treating Patients With Localized Kidney Cancer Undergoing Nephrectomy (PROSPER RCC)	Phase 3	Active, not recruiting	Neoadjuvant and adjuvant nivolumab & nephrectomy ± metastasectomy versus surgery alone	766	Event Free Survival	None Reported
Metastasectomy ± Adjuvant Therapy	NCT01444807	Evaluate the Efficacy of Sorafenib in Renal Cell Carcinoma Patients After a Radical Resection of the Metastases (RESORT Trial)	Phase 2	Active, Not recruiting	Adjuvant sorafenib vs. supportive care	132	Recurrence Free Survival	median RFS 37 months observation arm vs. 21 months in sorafenib arm (*p* = 0.404)
Metastasectomy ± Adjuvant Therapy	NCT01216371	Resection of Pulmonary Metastasis in Clear Cell Renal Cell Carcinoma +/-Adjuvant Sunitinib Therapy (SMAT)	Phase 2	Recruiting	Adjuvant sunitinib vs. placebo	60	2-year recurrence free survival	None Reported
Metastasectomy ± Adjuvant Therapy	NCT01575548	Pazopanib Hydrochloride in Treating Patients With Metastatic Kidney Cancer Who Have No Evidence of Disease After Surgery	Phase 3	Active, Not recruiting	Adjuvant pazopanib vs. placebo	129	Disease-free survival	Disease-Free Survival HR = 0.85
								*p* = 0.054
Metastasectomy ± Adjuvant Therapy	NCT03142334	Safety and Efficacy Study of Pembrolizumab (MK-3475) as Monotherapy in the Adjuvant Treatment of Renal Cell Carcinoma Post Nephrectomy (KEYNOTE-564)	Phase 3	Active, not recruiting	Nephrectomy ± metastasectomy with adjuvant pembrolizumab vs. placebo	994	Disease-free survival	No metastasectomy-specific results reported

OS, overall survival; RFS, recurrence-free survival; HR, hazard ratio.

## Conclusion

Complete response rate for mRCC continues to be low despite recent advancements in systemic therapy development. Metastasectomy has been demonstrated to be associated with improved clinical outcomes for mRCC. However, multiple factors affect these outcomes, including patient risk stratification, location of metastasis, synchronous or metachronous metastasis, and metastasis resectability. Additionally, there is a lack of RCTs exploring the benefit of metastasectomy for mRCC. Morbidity and mortality with metastasectomy are important considerations when determining which patients should undergo surgery, and complication types differ based on the site of metastasectomy. Furthermore, the decision to treat mRCC with metastasectomy should be guided by a multitude of factors, including patient performance status, age, disease progression, site of metastasis, and ultimately the patient's goals of care. Other prognostic factors including histologic and molecular analyses may further shape patient selection for metastasectomy as more research is conducted in this field. As our armamentarium of systemic therapies and ablative techniques continues to expand, it is important to continue evaluating if surgical resection of metastases offers a survival benefit and what the role of systemic therapy is after metastasectomy.
